# Supplementary figures and images for: Is it best to add native shrubs to a coastal sage scrub restoration project as seeds or as seedlings?
Source: PLoS One. 2022 Feb 8;17(2):e0262410. doi: 10.1371/journal.pone.0262410 (PMC8824352; doi:10.1371/journal.pone.0262410)

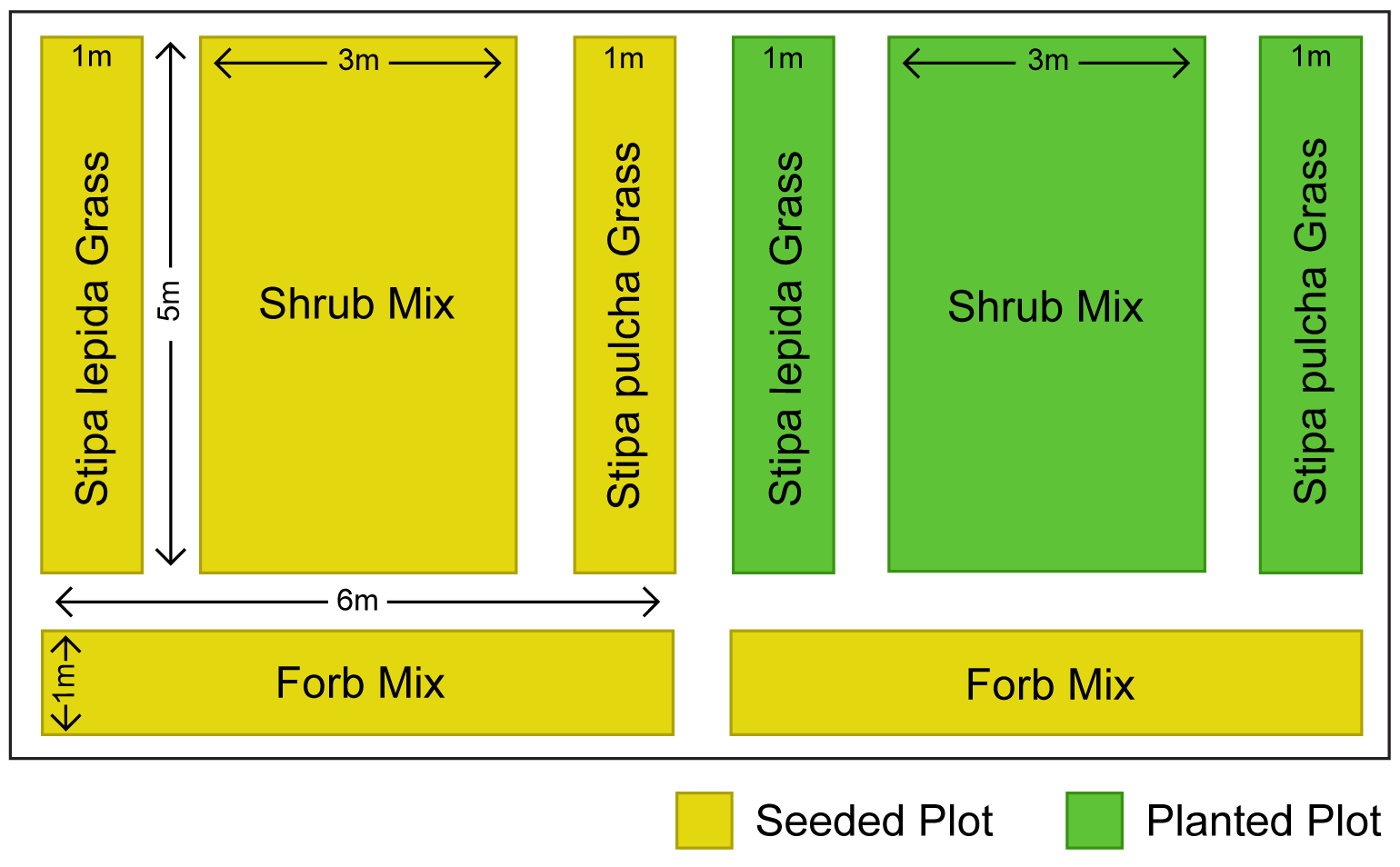

Supplement: S1 Fig — An example of an experimental block at the site. The block includes plots that accommodate all seed mixes, including two larger shrub mix plots, two forb plots, and four grass mix plots (two each for the two grassland species, Stipa pulchra and Stipa lepida, used in this study.) One half of the block was randomly assigned to the planting treatment, while the other half was seeded. Forb plots were seeded in both halves of the plots. (TIF) [file pone.0262410.s001.tif]

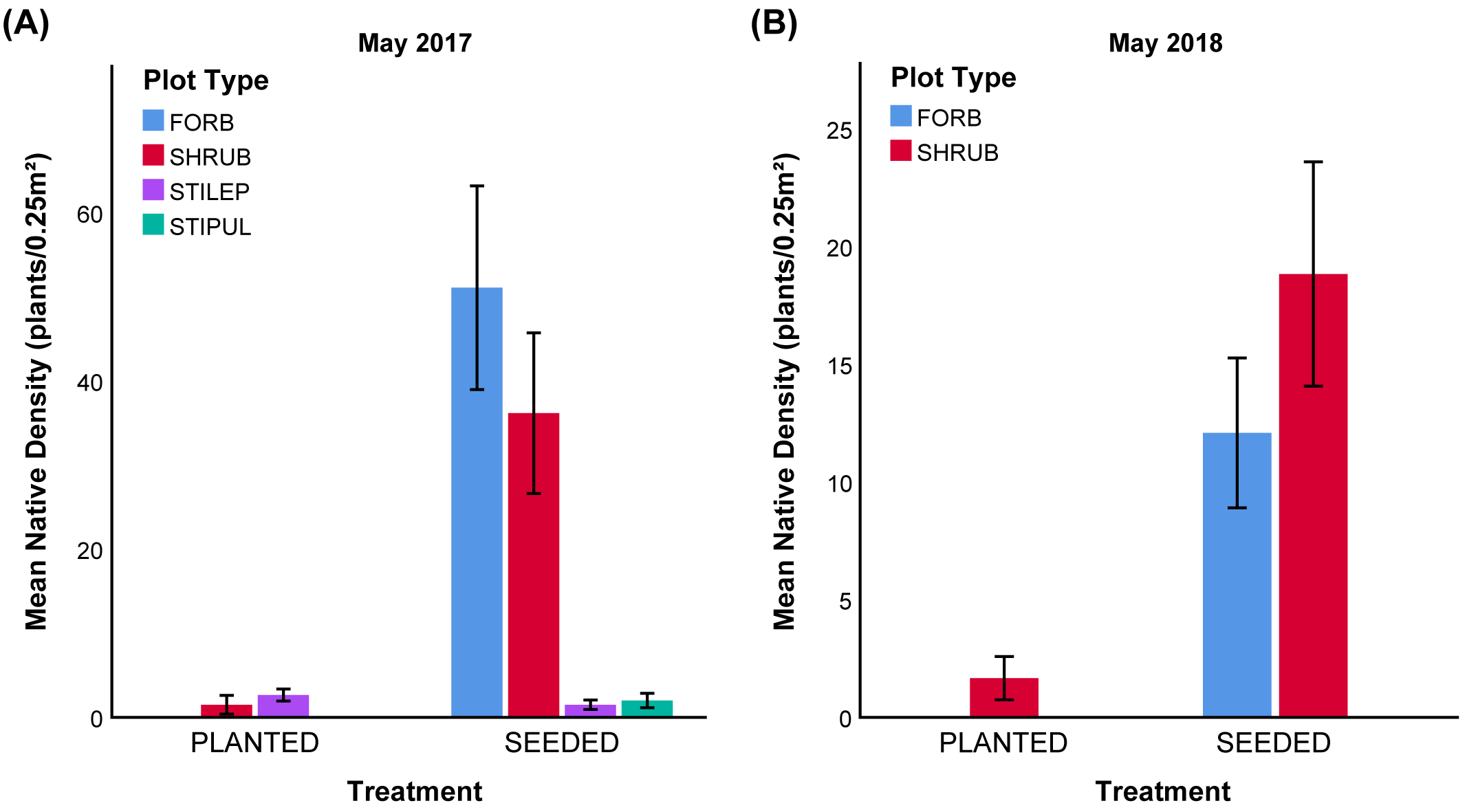

Supplement: S2 Fig — Density of plants seeded and planted into the respective plot types in A) May 2017 and B) May 2018. (Density data was not collected in the grass plots [STILEP and STIPUL] in May 2018. Bars represent mean +/− 1 SE. (TIF) [file pone.0262410.s002.tif]

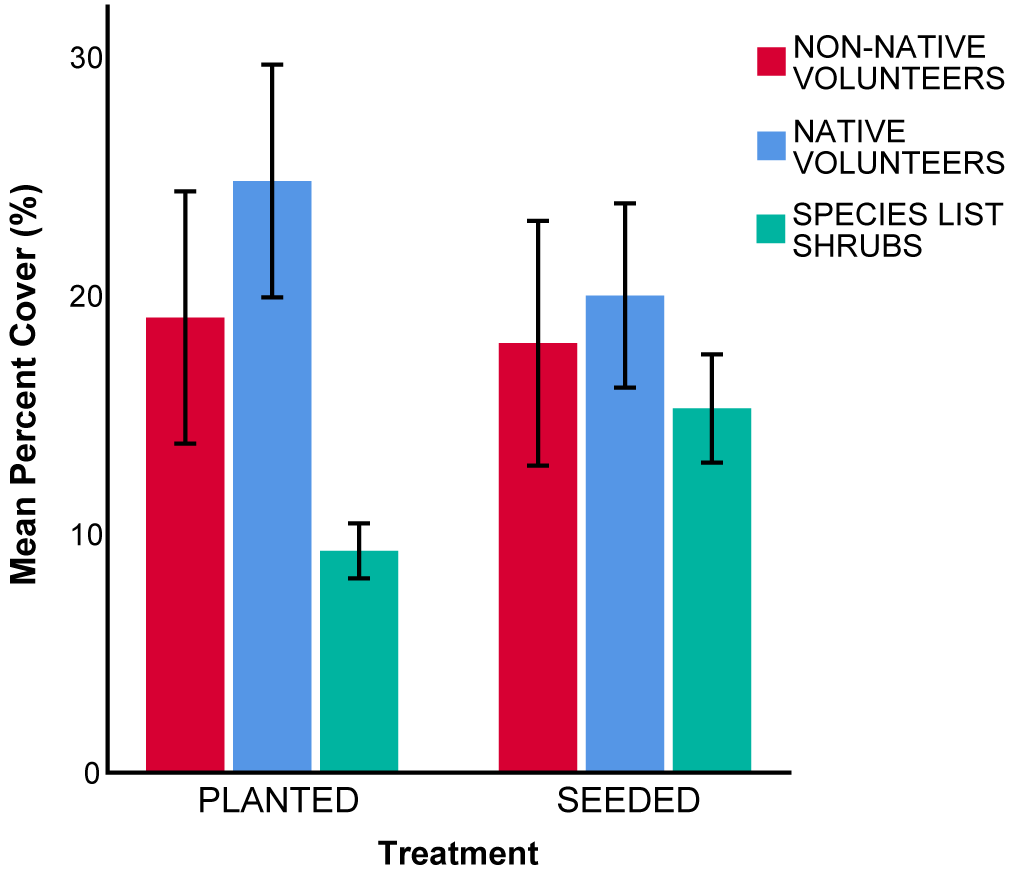

Supplement: S3 Fig — Mean percent cover of native plants that were on the shrub mix species list compared to volunteer plants that were not on the species list and established naturally. Volunteer plants are further broken down into natives and non-natives. Bars represent mean +/− 1 SE. (TIF) [file pone.0262410.s003.tif]

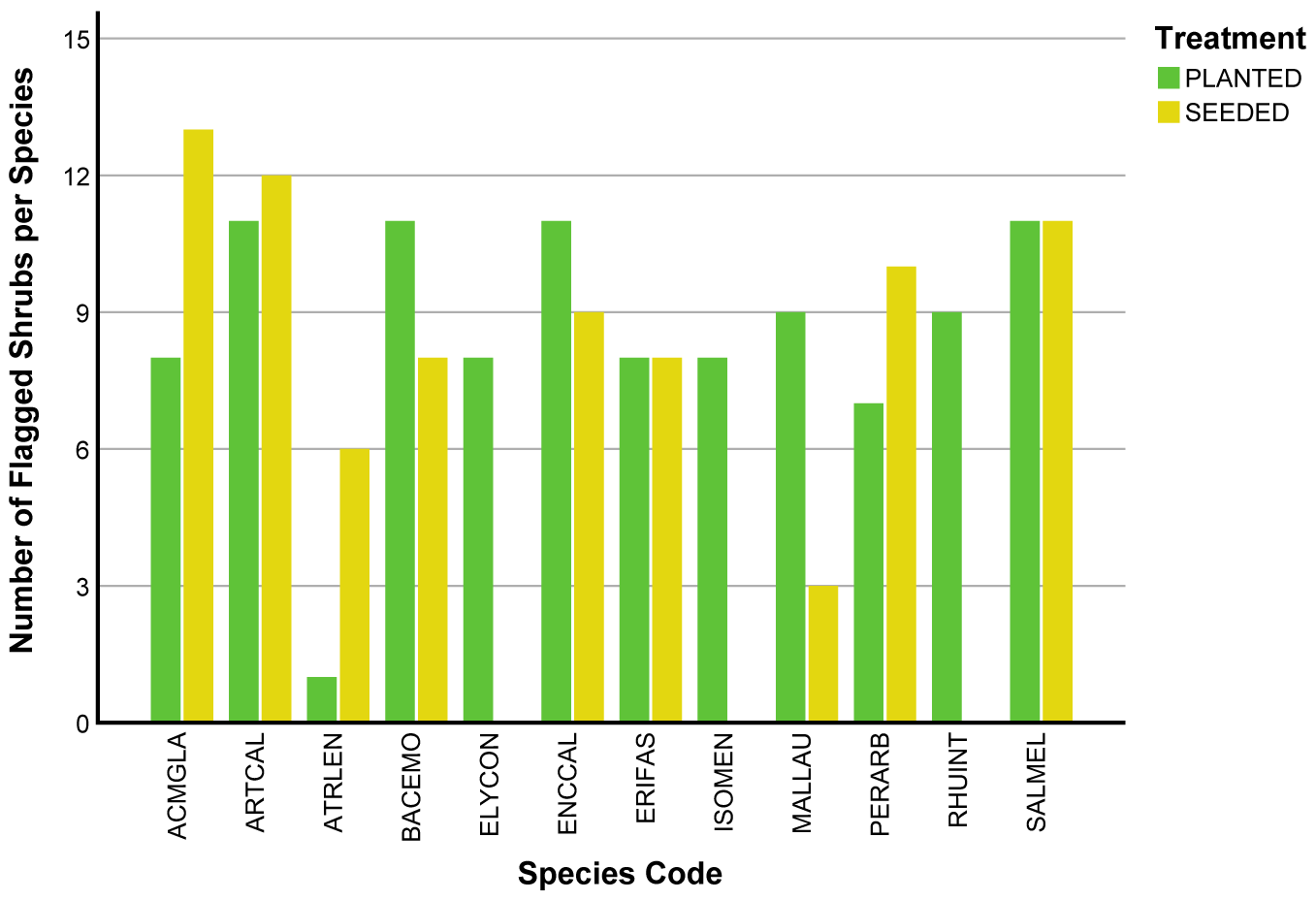

Supplement: S4 Fig — The number of tagged shrub mix individuals by species that were alive at the end of the study. (TIF) [file pone.0262410.s004.tif]
