# Supplementary material for: Is it best to add native shrubs to a coastal sage scrub restoration project as seeds or as seedlings?
Source: PLoS One. 2022 Feb 8;17(2):e0262410. doi: 10.1371/journal.pone.0262410 (PMC8824352; doi:10.1371/journal.pone.0262410)
Supplement: S1 Table — This table reports the percent pure live seed (PLS), seeding rate (seeds/m2), and dormancy breaking procedure used in preparation for seeding or planting efforts. Information on other restoration projects in the area was used to determine seeding rates. (DOCX) [file pone.0262410.s005.docx]

**S1 Table. Complete species list with associated seeding rates.**

| **Species** | | **Species Code** | **PLS (%)** | **Seeding Rate (seeds/m²)** | **Dormancy**  **Breaking Protocol** |
| --- | --- | --- | --- | --- | --- |
| **Shrub Mix** | Acmispon glaber | ACMGLA | 83 | 2072.9 | Scarify seed with blender |
|  | Artemisia californica | ARTCAL | 53 | 3035.8 |  |
|  | Atriplex lentiformis | ATRLEN | 73 | 542.7 |  |
|  | Baccharis emoryi | BACEMO | NA | NA |  |
|  | Elymus condensatus | ELYCON | 78 | 8.2 |  |
|  | Encelia californica | ENCCAL | 92 | 45.7 |  |
|  | Eriogonum fasciculatum | ERIFAS | 19 | 84.3 |  |
|  | Isocoma menziesii | ISOMEN | 28 | 118.5 |  |
|  | Malosma laurina | MALLAU | 94 | 210.9 | Cold stratify seed in the refrigerator for one month |
|  | Peritoma arborea | PERARB | 47 | 8.9 |  |
|  | Rhus integrifolia | RHUINT | 64 | 0.7 | Scarify seed with sandpaper and lightly score one side of the seed with a razor blade. Soak in warm water for four days |
|  | Salvia mellifera | SALMEL | 61 | 133.1 |  |
| **Grass Mix** | Stipa lepida | STILEP | 76 | 501.4 |  |
|  | Stipa pulchra | STIPUL | 74 | 236 |  |
|  | Deinandra fasciculata | DEIFAS | 78 | 607.2 |  |
| **Forb Mix** | Eschscholzia californica | ESCCAL | 85 | NA | Overnight soak in 5% liquid smoke solution |
|  | Phacelia cicutaria | PHACIC | 74 | 403.2 | Overnight soak in 5% liquid smoke solution |
|  | Salvia columbariae | SALCOL | NA | NA |  |
|  | Lupinus succulentus | LUPSUC | 84 | 24.5 | Overnight soak in hot water |
|  | Plantago erecta | PLAERE | 82 | 301.2 |  |
|  | Penstemon spectabilis | PENSPE | 59 | 2970.2 | Overnight soak in 5% gibberelic acid solution |
|  | Eriophyllum confertiflorum | ERICON | NA | NA |  |
|  | Amsinckia menziesii | AMSMEN | 68 | 136.7 |  |
|  | Lasthenia californica | LASCAL | 84 | 2316 |  |
|  | Lupinus bicolor | LUPBIC | 95 | 169 |  |

This table reports the percent pure live seed (PLS), seeding rate (seeds/m^2^), and dormancy breaking procedure used in preparation for seeding or planting efforts. Information on other restoration projects in the area was used to determine seeding rates.
