# Supplementary material for: Is it best to add native shrubs to a coastal sage scrub restoration project as seeds or as seedlings?
Source: PLoS One. 2022 Feb 8;17(2):e0262410. doi: 10.1371/journal.pone.0262410 (PMC8824352; doi:10.1371/journal.pone.0262410)
Supplement: S2 Table — Specific activities or materials associated with each generalized restoration task or purchase conducted in our study. (DOCX) [file pone.0262410.s006.docx]

**S2 Table. Description of restoration tasks.**

| **Generalized Restoration Task** | **Description** |
| --- | --- |
| Planting | Activities include transplanting container plants at the restoration site |
| Seed Mix Preparation | Activities include preparing native seed mixes and enhancing germination with species-specific dormancy breaking treatments |
| Seeding | Activities include sowing seed at the restoration site |
| Site Preparation & Maintenance | Activities include removing invasive, non-native plants |
| **Generalized Purchase Category** | **Description** |
| Contracted Grow-Out - Perennial | Nursery grown perennial plants |
| Perennial Grow-Out Supplies | Soil and potting materials |
| Perennial Seed Mix | Native perennial grass and shrub seed |

Specific activities or materials associated with each generalized restoration task or purchase conducted in our study.
