# Supplementary material for: Is it best to add native shrubs to a coastal sage scrub restoration project as seeds or as seedlings?
Source: PLoS One. 2022 Feb 8;17(2):e0262410. doi: 10.1371/journal.pone.0262410 (PMC8824352; doi:10.1371/journal.pone.0262410)
Supplement: Attachment — Submitted filename: Plos One.docx [file pone.0262410.s007.docx]

TITLE

The title is too generic for a study developed only in one site. It is necessary to clarify which types of native plants have been used (trees, shrub, grass…?) and which types of ecosystems have been restored (forests, savannas, grasslands…?).

ABSTRACT

Line 24 – It is necessary to clarify the term “species identity”. I do not think that the influence of the weather in the outcomes has been analyzed in the study.

Line 24 – The authors cited that that measured percent cover, density, diversity (probably they meant species richness), height… However, in the abstract they only showed and discussed the density and survival.

Line 32 – According to authors, the responses varied by species. The evaluation of species performances has not been cited as one of the objectives of the study. As a matter of fact, the objectives of the study are not clear in the abstract.

Line 34 – The conclusions and the objectives of the study are not properly linked. Moreover, the measurements made do not support this conclusion.

INTRODUCTION

Lines 47 – It is essential to explain which type of ecosystem the study aim to restore.

Lines 49 – 51 – This sentence is unnecessary, as the experiment did not study weed management.

Lines 55 – 56 – It is necessary to add a reference.

Lines 57 – 60 – It is necessary to add references. For example: Doust, S.J., Erskine, P.D., Lamb, D., 2006. Direct seeding to restore rainforest species: microsite effects on the early establishment and growth of rainforest tree seedlings on degraded land in the wet tropics of Australia. Forest Ecology and Management 234, 333-343; Doust, S.J., Erskine, P.D., Lamb, D., 2008. Restoring rainforest species by direct seeding: Tree seedling establishment and growth performance on degraded land in the wet tropics of Australia. Forest Ecology and Management 256, 1178-1188; Meli, P., Isernhagen, I., Brancalion, P.H.S., Isernhagen, E.C.C., Behling, M., Rodrigues, R.R., 2018. Optimizing seeding density of fast-growing native trees for restoring the Brazilian Atlantic Forest. Restoration Ecology 26, 212-219; Souza, D.C., Engel, V.L., 2018. Direct seeding reduces costs, but it is not promising for restoring tropical seasonal forests. Ecological Engineering 116, 35-44.

Lines 60 – 64 – These sentences are unnecessary, as the experiment did not study seed dormancy break.

Lines 66 – 68 – These sentences need references.

Line 76 – 77 – Add reference.

Lines 86 – 88 – Add reference.

Lines 89 – 93 – These sentences are unnecessary.

Lines 105 – 109 – This sentence is not clear. As a matter of fact, I think that the objectives section is too long, unclear, and a little disconnected to the methods. I suggest rewriting the entire section.

MATERIALS AND METHODS

Lines 122 – 123 – Add reference.

Lines 138 – 144 – This sentence is not clear.

Lines 145 – 150 – If different species was used in different years, the manuscript should not discuss the effects of weather on the outcomes.

Lines 179-180 – Only two individuals? That is a very small number of individuals.

Line 182 – Forbs were added just as seeds? So, there are no reasons to use them considering the main objective of the experiment.

Line 193 – If the data have not been included in the analyses, there are no reasons of cite it.

Line 198 – In the abstract and introduction sections the authors used the term “diversity” to refer to “species richness”. It is necessary to correct it.

Line 200 – Considering the objectives of the study, to evaluate the chlorophyll content is not necessary. This measurement was not important to discussion and conclusions.

Line 223 – I suggest eliminating this measurement.

RESULTS

Line 272 – It is not clear how seeded/planted individuals were differentiated from natural regeneration.

Line 282 – If the focus was on the shrub species, why to use forbs and grasses?

Line 291 – If the data is not statistical significant, it is not necessary to cite that they were higher or lower.

Line 295 – Explain “identity”.

Lines 338 – 340 – This is obvious. It is not necessary to present these results. I suggest removing this sentence and the next one.

DISCUSSION

Lines 403 – 405 – Your results do not support this affirmation.

Lines 409 – 411 – The measurement of chlorophyll content was not necessary.

Lines 414 – 416 – This was expected.

Lines 416 – 417 – Your results do not support this affirmation.

Line 422 – Diversity or species richness?

Line 425 – It is necessary to better explain the species-specific responses in the objectives, materials and methods, and results section.

Lines 453 – 455 – Add references. It is necessary to better explain this sentence.

Lines 457 – 458 – Your objectives and results are not clear about the evaluation of functional traits in your study. It is necessary to better explain it.

GENERAL OVERVIEW

The comparison of restoration efforts by seeds and seedlings is very important for restoration practitioners and the manuscript could improve the discussion. However, the manuscript is not clear and the objectives, results, discussion and conclusions are not entirely connected. To rewrite the objective section is essential, and also to better explains the results according to the objectives. There are several unnecessary sentences, while important analyses are not wee-explained. The manuscript only will be appropriate for publication after substantial changes.
