# Supplementary material for: Is it best to add native shrubs to a coastal sage scrub restoration project as seeds or as seedlings?
Source: PLoS One. 2022 Feb 8;17(2):e0262410. doi: 10.1371/journal.pone.0262410 (PMC8824352; doi:10.1371/journal.pone.0262410)
Supplement: Attachment — Submitted filename: Response to Reviewers.docx [file pone.0262410.s009.docx]

**RESPONSE TO ACADEMIC EDITOR**

Please review your reference list to ensure that it is complete and correct. If you have cited papers that have been retracted, please include the rationale for doing so in the manuscript text, or remove these references and replace them with relevant current references. Any changes to the reference list should be mentioned in the rebuttal letter that accompanies your revised manuscript. If you need to cite a retracted article, indicate the article’s retracted status in the References list and also include a citation and full reference for the retraction notice.

Response: To our knowledge, none of the references cited have been retracted.

P5 Please format Latin-scientific names. They should appear in italics.

Response: Thank you for catching this. The formatting has been corrected.

P5 L. 133 Seeds of some species….

Response: This sentence has been edited. It now reads:

“Seeds of some species (*Acmispon glaber, Eschscholzia californica, Lupinus bicolor, Lupinus succulentus, Malosma laurina, Penstemon spectabilis, Phacelia cicutaria, and Rhus integrifolia*) were subjected to dormancy-breaking techniques prior to seeding (S1 Table).”

P6L137, L139 and other places. Please avoid one-sentence paragraphs

Response: These single-sentences have been combined into a paragraph. It reads:

“In January 2017, all experimental plots were seeded and planted according to the randomized plot design. For seeding, we used hand-broadcasting, raking, and tamping, which has been shown to yield greater germination in coastal sage scrub communities compared to other methods [38].”

P6 It is still not clear to me how seeding/sowing was performed within each shrub plot. Did you mixed seeds of all species and sowed them randomly? Was it in a spatial regular pattern to reduce competition (as with planted seedlings)? What about grasses and forbs?

It is neither clear the reasons for including such different number of seeds/m2 per species in the shrub mix plots. Please add some references that support this particular selection.

Please describe with more detail your sowing/seeding method and the reasons for including those different seeding rates per species. This is of particular importance as inn the discussion it is included that (L365) “This, as well as the greater mortality of seeded plants, could be due to high seeding rates. Competition of many emerging plants packed closely together may have limited plant growth and lowered growth and survival in the seeded treatment [83]. Determining seeding rates that are high enough to fill open space without leading to decreased growth due to competitive interactions is one of the complexities of conducting restoration from seed [24].”

Response: The following sentences have been added to further specify the seeding technique:

“Seeds of all shrub, grass, and forb species were combined into their respective seed mixes. The seeding rates varied greatly between different species, as is common in coastal sage scrub restoration [36,37]. Due to previous difficulties with restoring the site, we chose to use higher seeding rates than are typical. The seeding rate for *Acmispon glaber* is unusually high due in part to a calculation error, in which the intended seeding rate was doubled.”

“Seed mixes were sprinkled onto the ground by hand, using no specific spatial pattern, while taking care to distribute the seed evenly throughout each plot.”

To address the differences in seeding rates for the different species, the following references have been added:

Aprahamian AM, Lulow ME, Major MR, Balazs KR, Treseder KK, Maltz MR. Arbuscular mycorrhizal inoculation in coastal sage scrub restoration. Botany. 2016 Jun;94(6):493–9.

Tamura N, Lulow ME, Halsch CA, Major MR, Balazs KR, Austin P, et al. Effectiveness of seed sowing techniques for sloped restoration sites. Restoration Ecology. 2017;25(6):942–52.

P6L152 “to decrease plant competition” Otherwise include a reference that backs up the fact that at those distances among planted individuals there is no plant competition.

Response: This sentence has been rephrased. It now reads:

“We spaced out all the plants within their plot boundaries to decrease plant competition.”

Table 2. I would suggest highlighting in bold those treatments that render significant results (this is more common that highlighting in italics those that are not significant).

Response: Thank you for the suggestion. Significant results are now shown in bold.

**RESPONSE TO REVIEWER 2**

Reviewer #2: This revision addresses most of the concerns from the prior review; however, several new issues need attention. One major issue that the authors should address is that the species that drove the patterns in the seeded treatment (Acmispon glaber) was seeded at a much higher seeding rate than all other species (over 2000 seeds/m2 compared to less than 100 seeds for some other species). Because of this, the results really just show the effect of the treatments on this species. I think a caveat explaining this should be added to the discussion, and some justification for the discrepancy in seeding rates among species should be provided.

This is a very valid point. We reviewed our original seeding rate calculations for the study and found the cause of this discrepancy. In the Materials and Methods section, we explain that the seeding rate for all species are intentionally higher than usual due to previous difficulties restoring the site. We also explain that the rate for *Acmispon glaber* “is unusually high due in part to a calculation error, in which the intended seeding rate was doubled.”

The Discussion section has been revised to note this as well. It explains:

“Seeded plots outperformed planted plots in terms of native shrub density, but this trend was driven by one species, *Acmispon glaber*.”

and

“The two species that established best from seed in our study, *Atriplex lentiformis* and *Acmispon glaber*, are both drought-deciduous at our site, and they are both known to colonize areas from seed following wildfires [90,91]. *A. glaber* had a greater density and percent cover than any other species in the seeded treatment, likely due to its unintentionally high seeding rate. This species drove the trends in seeded plot cover and density. Its role in the coastal sage scrub community as an important early-successional fire-follower [92] and as a species that grows quickly in recently cleared shrublands [93] may have also contributed to *A. glaber*’s success when seeded. Further studies could investigate this possibility.”

To address the differences in seeding rates for the different species, references have been added. These show that using greatly different seeding rates for coastal sage scrub species is common in restoration projects.

Aprahamian AM, Lulow ME, Major MR, Balazs KR, Treseder KK, Maltz MR. Arbuscular mycorrhizal inoculation in coastal sage scrub restoration. Botany. 2016 Jun;94(6):493–9.

Tamura N, Lulow ME, Halsch CA, Major MR, Balazs KR, Austin P, et al. Effectiveness of seed sowing techniques for sloped restoration sites. Restoration Ecology. 2017;25(6):942–52.

68 Correct typo “success restoration success”

Response: This typo has been corrected.

74 The word “harsh” is subjective. Consider removing.

Response: As suggested, the word “harsh” has been removed. The sentence now reads:

“For one, using container plants is generally much more expensive than purchasing seeds [13], which places limits on the quality of restoration that can be achieved with a given budget.”

89 “We hypothesized that container plants would be best for increasing cover, while seeds would be best for increasing richness and that the plants grown from seed might grow faster and have higher survivorship due to improved root health.” These hypotheses seem to come out of the blue here. They need to be introduced earlier in the introduction. It is not obvious why container plants would be best for cover, but seeds would be best for richness. Is it because you can also add annuals as seed? Why would plants grown as seed have healthier roots than container plants? This was not introduced. Please provide some background to support the hypotheses.

Response: This section has been revised to better explain our reasoning. It reads:

“We hypothesized that container plants would be best for increasing cover because they are larger when added to the site, while seeds are generally better for increasing richness because they allow for annuals to be added. We hypothesized that seeded plots would have more unique species growing in a standardized area due to smaller stature of seedlings that germinated in the field. Plants grown from seed might grow faster and have higher survivorship due to improved root health [32,33]. We also hypothesized that restoration from seed would be less expensive and time-consuming than restoration from container plants [5]. Finally, we did not have any reason to expect the plants’ success to vary significantly by species or functional traits.”

100, 102, etc. On line 100 South is capitalized. On line 102 north is not capitalized. Please be consistent throughout the paper.

Response: All instances of cardinal directions have been decapitalized.

137-140 Combine these stray sentences into one of the paragraphs.

Response: These single-sentences have been combined into a paragraph. It reads:

“In January 2017, all experimental plots were seeded and planted according to the randomized plot design. For seeding, we used hand-broadcasting, raking, and tamping, which has been shown to yield greater germination in coastal sage scrub communities compared to other methods [38].”

150-151 “Between 47 and 69 of each Stipa pulchra and Stipa lepida individuals were planted in their respective plots.” Does this mean 47 S. pulchra and 69 S. lepida? Or were the numbers variable within each species? Please revise for clarity.

Response: To improve clarity, the sentence now reads:

“Between 47 and 69 *Stipa* individuals were added to each planted-treatment grass plot.”

151 Provide a reference for the planting density to show there was no competition. Otherwise you should not infer there was no competition without measuring it in the study.

Response: This sentence has been rephrased. It now reads:

“We spaced out all the plants within their plot boundaries to decrease plant competition.”

156 I think you can remove the heading “Maintenance” as this is just one sentence that can be included at the end of the prior section.

Response: The heading “Maintenance” has been removed.

220 List all the factors and interaction terms for each model. Otherwise it is not clear. Did the repeated measures model also include fixed factors? Which interaction terms were tested in each model? If no interaction terms were included why was this decision made? The logistic regression model does not list any factors at all. It seems that species x treatment should be included in all models with species as a factor since that was one of the reasons for the experimental design. Perhaps referring to Table 1 which shows the fixed factors for each model would be sufficient.

Response: We agree that this was confusing, and we have revised the data analysis section of the Methods to clarify the factors included in each model, as recommended. In particular, we were careful to include all factors included in each model, and to clarify why interactions were included.  Note that species was not included when models were based on more holistic measures of restoration success, such as native plant species richness.

Table 1 – The information in this table is helpful for interpreting the results; however, the formatting as one large table is kind of unusual. I suggest looking in the journal for other examples and formatting the table in a similar way. I also suggest reducing the number of decimal places to make it easier to read.

Response: This table has been reformatted into three sections - one for ANOVA, one for Repeated-Measures ANOVA, and one for Logistic Regression. To improve readability, cells containing the same dependent variable have been merged. When possible values were rounded to three decimal places.

We believe that the use of a large table to report the statistical results is appropriate. We referenced several other PLOS ONE articles to match the formatting as best as possible.

Similar tables are present in the following PLOS articles:

- [Changes in Soil Carbon and Nitrogen following Land Abandonment of Farmland on the Loess Plateau, China](https://journals.plos.org/plosone/article?id=10.1371/journal.pone.0071923)
- [Seasonal Dynamics of the Plant Community and Soil Seed Bank along a Successional Gradient in a Subalpine Meadow on the Tibetan Plateau](https://journals.plos.org/plosone/article?id=10.1371/journal.pone.0080220)
- [Effects of 10-Year Management Regimes on the Soil Seed Bank in Saline-Alkaline Grassland](https://journals.plos.org/plosone/article?id=10.1371/journal.pone.0122319)
- [Restoration management of cattle resting place in mountain grassland](https://journals.plos.org/plosone/article?id=10.1371/journal.pone.0249445)

Results – When an interaction term is significant it is best to interpret only the interaction term and not the individual factors. For example, if species x treatment is significant tell us which species were greater in seeded and which were greater in planted and which showed no effect of treatment, but there is no need to also discuss the overall differences among seeded versus planted. This approach should be applied to cover of native shrubs and lifespan.

Response: The suggested edits have been made. These sections read:

“*Acmispon glaber*, *Atriplex lentiformis*, *Encelia Californica*, *Eriogonum fasciculatum*, and *Salvia mellifera* had greater cover when seeded. *Elymus condensatus, Isocoma menziesii, Malosma laurina,* and *Rhus integrifolia* had greater cover when planted, but very low cover in both treatments. Cover of *Artemisia californica, Baccharis emoryi,* and *Peritoma arborea* showed no effect of treatment”

“There was a significant treatment-by-species interaction for shrub lifespan. *Acmispon glaber* and *Atriplex lentiformis* lived longer when seeded. *Artemisia californica*, *Baccharis emoryi*, *Encelia californica*, *Eriogonum fasciculatum*, *Malosma laurina*, *Peritoma arborea*, and *Salvia mellifera* had longer lifespans when planted. The remaining species did not have enough surviving individuals to compare.”

Table 3 This table should indicate that the costs for nursery-grown plants are estimated since they were not used in this study.

Response: The following sentence has been added to the description for Table 3:

“It also estimates the cost of using nursery-grown plants.”

363 Competition is one possibility for higher mortality, but so is desiccation stress during establishment. Seedlings in the seeded plots were smaller with less developed root systems and may not have tolerated stress as well as the planted shrubs. This is a pretty well-established barrier to restoration in most systems and is a reason why planted shrubs are used.

Response: Thank you for bringing up this point. This has now been mentioned in the discussion section:

“The higher mortality of seeded shrubs may also be because they were younger and less developed, and therefore may not have been able to tolerate environmental stress as well as their planted counterparts [87].”
